# *De Novo* Transcriptome Dataset Generation of the Swamp Buffalo Brain and Non-Brain Tissues

**DOI:** 10.1155/2022/4472940

**Published:** 2022-10-12

**Authors:** Wang Xiaobo, Faiz-ul Hassan, Sheng Liu, Shuli Yang, Muhammad Ahmad, Ishtiaq Ahmed, Kongwei Huang, Hafiz M. N. Iqbal, Hui Yu, Qingyou Liu, Saif ur Rehman

**Affiliations:** ^1^State Key Laboratory for Conservation and Utilization of Subtropical Agro-Bioresources, Guangxi University, Nanning 530005, China; ^2^Guangdong Provincial Key Laboratory of Animal Molecular Design and Precise Breeding, School of Life Science and Engineering, Foshan University, Foshan 528225, China; ^3^Institute of Animal and Dairy Sciences, Faculty of Animal Husbandry, University of Agriculture, Faisalabad 38040, Pakistan; ^4^Faculty of Veterinary Sciences, Shaheed Benazir Bhutto University of Veterinary and Animal Sciences (SBBUVAS), Sakrand 67210, Pakistan; ^5^Department of Regional Science Operations, La Trobe Rural Health School, Albury-Wodonga, Victoria 3690, Australia; ^6^Tecnológico de Monterrey, School of Engineering and Sciences, Monterrey 64849, Mexico

## Abstract

The sequenced data availability opened new horizons related to buffalo genetic control of economic traits and genomic diversity. The visceral organs (brain, liver, etc.) significantly involved in energy metabolism, docility, or social interactions. We performed swamp buffalo transcriptomic profiling of 24 different tissues (brain and non-brain) to identify novel transcripts and analyzed the differentially expressed genes (DEGs) of brain vs. non-brain tissues with their functional annotation. We obtained 178.57 Gb clean transcriptomic data with GC contents 52.77%, reference genome alignment 95.36%, exonic coverage 88.49%. Totally, 26363 mRNAs transcripts including 5574 novel genes were obtained. Further, 7194 transcripts were detected as DEGs by comparing brain vs. non-brain tissues group, of which 3,999 were upregulated and 3,195 downregulated. These DEGs were functionally associated with cellular metabolic activities, signal transduction, cytoprotection, and structural and binding activities. The related functional pathways included cancer pathway, PI3k-Akt signaling, axon guidance, JAK-STAT signaling, basic cellular metabolism, thermogenesis, and oxidative phosphorylation. Our study provides an in-depth understanding of swamp buffalo transcriptomic data including DEGs potentially involved in basic cellular activities and development that helped to maintain their working capacity and social interaction with humans, and also, helpful to disclose the genetic architecture of different phenotypic traits and their gene expression regulation.

## 1. Introduction

The buffalo belongs to the family *Bovidae* (genus *Bubalus*) and is considered a significant livestock species owing to its multiple utilities as a source of meat, milk, and draught power in agricultural fields [1–3]. Buffaloes are usually found in wet grasslands, swamps, and marshes, subtropical and tropical regions of the world. The Asian domesticated water buffalo is generally categorized into two main subspecies including the swamp (2*n* = 48) and river buffalo (2*n* = 50) usually based on their physical appearance, body size, chromosome karyotype, and physiological features [2, 4, 5]. In China, swamp buffaloes are native animals distributed across 18 provinces in southern and central China. Based on their local regional distribution, these buffaloes have been grouped into 18 local breeds [6, 7]. Swamp buffaloes were mostly reared by small farmers as a draught power for agricultural operations, particularly ploughing in rice paddy fields. However, owing to its economic traits like leather, horns, meat, and milk, over the last decade, extensive efforts have been made for the genetic improvement of dairy traits in buffalo through crossbreeding [2, 8, 9].

The major impediment in China's buffalo industry included poor reproductive performance and milk production of local buffalo as compared to dairy cattle, so major efforts were directed towards improving the buffalo herd size to increase reproductive efficiency through utilizing reproductive technologies [8, 10–13], to identify the genetic markers and genes, which were associated with phenotypic variations [14–16] of desirable traits [17–19]. In China, the information related to buffalo breeding is still limited regarding molecular breeding techniques. The lacking of genomic information is the key hindrance in buffalo genetic improvement programs, although several studies at the genomic level have been conducted so far by different research groups [20–22].

Even though the draft genome of the swamp and river buffalo has been released [23, 24], but genetic information on different physiological traits of buffalo is still scanty which in turn hinders the buffalo's genetic improvement [25, 26]. The transcriptomic studies are important to generate larger quantities of sequenced data for both model and non-model species [27]. In different species like sheep [28, 29], goat [30] cattle [31], and pig [32], high-throughput technologies such as RNA sequencing (RNA-seq) have efficiently been used in transcriptome analysis, molecular marker development, and gene discovery.

The swamp buffaloes have shown closer association with humans mainly because of their key utility as a draft power in agroecosystems. The genetic basis of this close social interaction of swamp buffalo has also been revealed at the genomic level in a recent study [23] that explained the selection signatures for social behavior and energy related genes in the swamp buffalo, which facilitated them to develop long-term collaboration with humans in rice paddy field work. Further, the visceral organs, like the brain, liver, heart, lungs, spleen, and kidney, etc. are the key organs that play a significant role in energy metabolism, docility, and/or social interactions. It is therefore imperative to explore the differential expression of genes associated with physiological responses and neural networks to better understand adaptive and cognitive behaviors. This study was designed with the aim to perform the transcriptomic profiling of 24 different tissues of swamp buffalo (grouped into brain and non-brain tissues), to analyze the DEGs, to evaluate the novel transcripts, and their functional annotation.

## 2. Materials and Methods

### 2.1. Sample Collection and Preparation

An adult female swamp buffalo, which was kept under uniform feeding conditions without any biotic or abiotic stress, was purchased from SIYE buffalo farm Guanxi, China, for slaughtering and sample collection. A total of 24 samples from different body parts of the swamp buffalo were collected. These samples were categorized into two groups, including the brain and non-brain tissues. The details of the samples are given in Table 1. All these samples were used for transcriptomic sequencing analysis.

### 2.2. RNA Extraction, Quantification, and Quality Assessment

The total RNA of each sample was extracted by using the Trizol method [33]. Further, the purity and concentration of RNA were checked by using NanoDrop 2000 (Thermo Fisher Scientific, Wilmington, DE), and the integrity of RNA was evaluated through the RNA Nano 6000 Assay Kit of the Agilent Bioanalyzer 2100 system (Agilent Technologies, CA, USA).

### 2.3. Library Preparation for Transcriptomic Sequencing

To prepare the RNA sample, 1 *μ*g RNA from each sample was used. The NEBNext UltraTM RNA Library Prep Kit for Illumina (NEB, USA) was used to generate the sequence libraries by following the recommendations of the manufacturer, and index codes were given to each sample feature. Briefly, the magnetic beads (poly-T oligo-attached) were used to purify the mRNA from total RNA. In NEBNext, first-strand synthesis reaction buffer (5×) at high temperature divalent cations was used for disintegration. The first cDNA strand was produced by using a random hexamer primer along with M-MuLV Reverse Transcriptase, while RNase H and DNA polymerase I was subsequently used to synthesize the second cDNA strand. The remaining overhangs via exonuclease/polymerase activities were changed into blunt ends. After the adenylation of DNA fragments 3′ ends, the hairpin loop structure and NEBNext adaptor were ligated for hybridization purposes. The AMPure XP system (Beckman Coulter, Beverly, USA) was used to purify the library fragments to select cDNA fragments especially in the length of 240 bp. Meanwhile, before PCR, a 3 *μ*l of USER Enzyme (NEB, USA) was added with selected size and ligated-adaptor to cDNA at 37°C for 15 minutes and followed by 5 minutes at 95°C. Then, universal PCR primers, Index (X) primer, and Phusion High-Fidelity DNA polymerase were used to perform the PCR. At last, the AMPure XP system was used to purify the PCR products, and Agilent Bioanalyzer 2100 system was employed to access the quality of the library [34].

### 2.4. Clustering and Sequencing

The cBot Cluster Generation System using TruSeq PE Cluster Kit v4-cBot-HS (Illumina) was used to perform the index-coded samples clustering analysis as per the manufacturer's instructions. After the generation of the cluster, the prepared library was sequenced by using an Illumina platform (HiSeq X Ten), and reads with paired ends were produced.

### 2.5. Data Analysis

#### 2.5.1. Quality Control

Firstly, the in-house Perl scripts were used to process the raw reads (raw data). The clean reads (clean data) were obtained after removing the reads having ploy-N and low-quality and adaptor sequences from the raw data. Moreover, the GC-content, Q20, Q30, and level of sequence duplication in clean reads were calculated. The high-quality clean data was used for further downstream analyses [35].

#### 2.5.2. Comparative Analysis

Subsequently removing the low-quality and adaptor sequences from the data sets, the clean reads after data processing were transformed from raw sequences. Hisat2 tools software was used to map the clean reads to the reference genome and the sequences with exact match or single mismatch were further evaluated and annotated to the reference genome.

#### 2.5.3. Gene Functional Annotation

For gene functional annotation, various databases were utilized including, Nt (NCBI nonredundant nucleotide sequences), Nr (NCBI nonredundant protein sequences), KOG/COG (Clusters of Orthologous Groups of proteins), Pfam (Protein family), GO (Gene Ontology), Swiss-Prot (A manually annotated and reviewed protein sequence database), and KO (KEGG Ortholog database) [36, 37].

#### 2.5.4. SNP Calling

For each sample sorting, removing the duplicated reads and bam alignment merging was done by samtools (v0.1.18) and Picard-tools (v1.41). Moreover, SNP calling was accessed by GATK2 or samtools software. The GATK standard filter method with other parameters (including cluster Window Size: 10; MQ0 > = 4 and (MQ0/(1.0^∗^DP) > 0.1; QUAL < 10; QUAL < 30.0 or QD < 5.0 or HRun > 5), were used to filter the raw vcf files and the SNPs with distance > 5 were retained [35, 38].

#### 2.5.5. Quantification of Gene Expression Levels

The levels of gene expression were predicted in fragments per kilobase of transcript per million fragments mapped (FPKM) value by using the following formula:

(1) $$\begin{array}{c} \text{FPKM}=\frac{\text{cDNA fragments mapped}}{\text{fragments }\left(\text{millions}\right)}\times \text{transcript length }\left(\text{kb}\right). \end{array}$$

#### 2.5.6. Differential Expression Analysis

*(1) For the Samples with Biological Replicates*. The DESeq2 was used to analyze the differential expression of the two tissue groups. Based on the negative binomial distribution model, DESeq2 provided practices to determine the differential expression of the digital gene expression dataset. Benjamini and Hochberg's approach were used to adjust the *P* value to control the false discovery rate (FDR). Statistically, the *P*value < 0.05 was used as the level of significance, and the genes with *P*value < 0.05 were perused as differentially expressed [39, 40].

*(2) For the Samples without Biological Replicates*. For two samples, the edgeR was used to analyze the differential expression and the FDR value < 0.05 (FDR < 0.05) and fold change ≥ 2 (FC ≥ 2) was set as a criteria to categorize the significant differential expression [40, 41].

*(3) GO and KEGG Pathway Enrichment Analysis*. The Wallenius noncentral hypergeometric distribution based GOseq R packages [42] were used for GO (Gene Ontology) enrichment analysis of DEGs. The KEGG [43] is a biological system related database resource used to understand high-level utilities and functions associated with cells or organisms at the molecular level especially the large scale molecular datasets developed by high-throughput genome sequencing and experimental technologies (http://www.genome.jp/kegg/). The KOBAS [44] software was used to test the statistical enrichment of DEGs in KEGG pathways [43].

## 3. Results

### 3.1. Quality Assessment of the Data

#### 3.1.1. The Sequence Quality Score with Content Distribution and Data Statistics

The quality of the data was accessed by using Phred quality scores *Q* ,which is logarithmically associated with base calling error probabilities (*P*). The quality of all sample data with base error rates and ATCG content distribution is presented in Figure S1. All the samples showed an equal distribution of ATCG content revealing the accuracy of the data (Figure S1). Furthermore, after the quality control of sequenced data, a total of 178.57 Gb clean data were obtained with a minimum *Q* score as ≥ Q30 (91.27%) represented a 1/1000 probability of incorrect base call. The higher *Q* value results in lowering the false positive variants with the more consistent and reliable data set. The clean reads, clean bases, and GC contents were ranged between 21868067-28337044, 6560420100-8501113200, and 49.47-52.77%, respectively (Table S1).

#### 3.1.2. The Transcriptomic Data Alignment with Reference Genome Sequence

Clean data read without paired ends were mapped to the reference genome exhibiting an alignment percentage (%) between 89.53% and 95.36% (Table S2). While the unique mapped read coverage was 84.88% to 92.48%, and the clean read percentage which multiply mapped to the reference genome was 2.13% to 4.83% (Table S2). Whereas, the percentage of clean reads marked on the sense vs. antisense chain of the reference genome was 44.58% vs. 43.79% to 47.50% vs. 47.47% (Table S2).

#### 3.1.3. The Mapped Data Distribution on the Reference Genome with Exon, Intron, and Intergenic Regions

Additionally, the genome wide distribution of the reader's coverage was retrieved to find the location and distribution of the mapped reads on different chromosomes in terms of coverage depth, plotted on the reference genome with log2 value ranged between −10 and 10 (Figure S2). The blue and green color represents the reads coverage on the positive and negative chain of the reference genome, respectively (Figure S2). Moreover, for each sample type the percentage of different regions including intronic, exonic, and intergenic regions based on number of mapped reads in reference to the specified reference genome were counted (Table S3). The highest exon count percentage was observed as 88.49% in the LC sample and the overall range was between 75.81% and 88.49% (Table S3). The percentage of intronic and intergenic regions for all samples was 5.05% to 16.74% and 6.04% to 7.89%, respectively (Table S3).

#### 3.1.4. The RNA-Seq Library-Quality Evaluation

The RNA-seq library quality was accessed employing transcripts depth coverage to evaluate the randomness of the mRNA degradation and mRNA fragmentation, the distance from paired-end of read1 and read2 to judge the inserted lengths distribution extent, and the data saturation to assess the library capacity and mapped data adequacy (Figure S3(a) and (b)). All the sample RNA fragments' randomness was observed uniformly, which was simulated based on the density of mapped reads on transcripts as shown in Figure S3(a). Further, for each sample data, the gene saturation with an interval of 15% FPKM was observed, and a gradual increase was seen, with gene saturation detected as 1 (Figure S3(b)).

### 3.2. Single Nucleotide Polymorphisms/InDel Analysis

Single nucleotide polymorphism (SNP) is referred to a single nucleotide variation in transcript sequence. We used GATK to identify the single base mismatch between the sample transcripts and the reference genome as a potential SNP site. The higher number of SNPs was perceived in *P* (495,289) and the lower number was detected in YD44-45Q4 (183,666) (Table S4). The spotted genic and intergenic SNPs were ranged between 153151 to 456941 and 29406 to 68294, respectively (Table S4). A higher ratio of transitions SNPs (*A* > *G*, *G* > *A*, *C* > *T*, and *T* > *C*) with a percentage between 71% and 73.22% as compared to the transversions (*A* > *C*, *C* > *A*, *A* > *T*, *T* > *A*, *C* > *G*, *G* > *C*, *G* > *T*, and *T* > *G*) was detected in all transcriptomic data (Table S4, Figure S4). Moreover, the SNP sites heterozygosity (more alleles) proportion was also determined which ranged from 20.31% in QS to 25.04% in KJ24-32Q (Table S4). The SNPs density for all the samples is presented in Figure S5, which showed a gradual increase of SNPs per kb of the gene length. But, the number of genes was inversely proportional to the number of SNPs per Kb (Figure S5).

**Figure 1:**
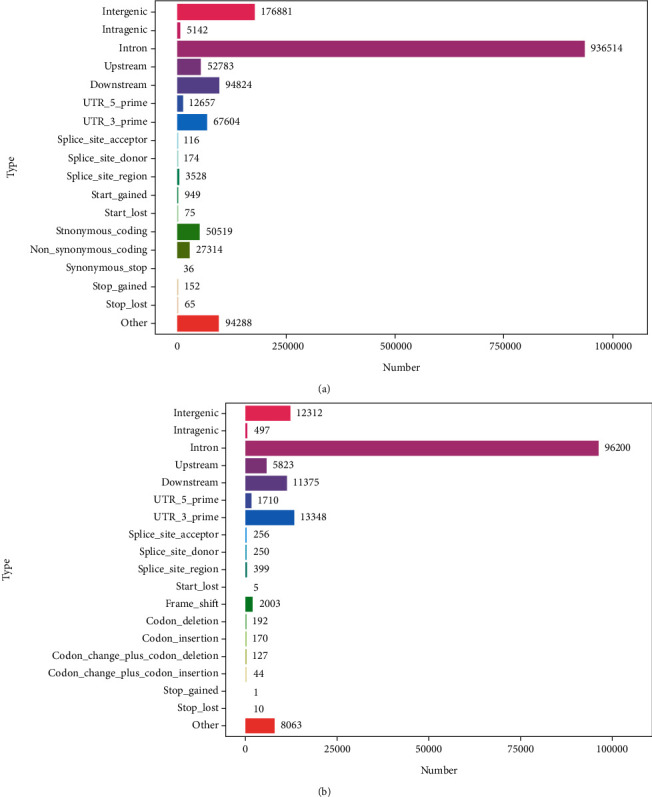
The annotation classification of (a) SNPs and (b) InDel. [Note: The abscissa represents SNPs and InDel areas or types while ordinate is the classification numbers].

Furthermore, the SnpEff tool was used to predict the SNP and InDel variability impact. In reference to the position and information on the reference genome, the location of variable sites in reference genome regions (CDS, intergenic, or genic regions, etc.) and their potential effects (nonsynonymous or synonymous mutations) were obtained (Figures 1(a) and 1(b)). A total of 936514, 176881, 94824, 67604, 52783, 12657, and 5142 SNPs were found in intronic, intergenic, downstream, 3′UTR, upstream, 5′UTR, and intragenic regions, respectively (Figure 1(a)). The synonymous coding/nonsynonymous coding SNPs ratio was 50519/27314 (Figure 1(a)). Besides, the annotated InDel retrieved on the reference genome were 96200, 13348, 12312, 11375, and 5823 in the intron, 3′UTR, intergenic, downstream, and upstream regions, respectively (Figure 1(b)).

The alternative splicing events for all samples were scanned by the ASProfile tool, which divided all these events into 12 different types. The TSS, TTS, AE, and SKIP were the most abundant mapped alternative splicing events of which the first alternative 5′ exon splicing (TSS) and alternative 3′ last exon splicing (TTS) were highly screened in all samples with value > 15000 (Figure S6), while XMIR was not detected in LC, P, QG23-31, QG35Q, SGT, WJYY43Q, and XN samples (Figure S6). Except for sample F, P, QS, S, YQZ, ZF, and ZG, the lower alternative splicing events XAE and XIR were also identified in all samples (Figure S6). While an equal ratio of XSKIP event was detected in all samples (Figure S6).

### 3.3. Novel Genes Detection and Functional Annotation

We used string tie to assemble the mapped reads based on the referenced genome and the original genome annotation was compared to discover the unique unannotated transcriptional regions, revealing novel transcripts and genes in the buffalo, and improved the existing genome annotation information. A total of 5,574 novel genes were discovered after filtering the short peptides (<50 amino acid) and the sequence with a single exon. All the novel genes were blasted in different databases including GO, KEGG, KOG, Pfam, Swiss-Prot, eggNOG, NR, and COG to obtain the annotation information. The novel gene number annotated by different databases is summarized in Table 2.

### 3.4. Analysis of Genes Expression

#### 3.4.1. Quantitation of Gene's Expression Levels

**Figure 2:**
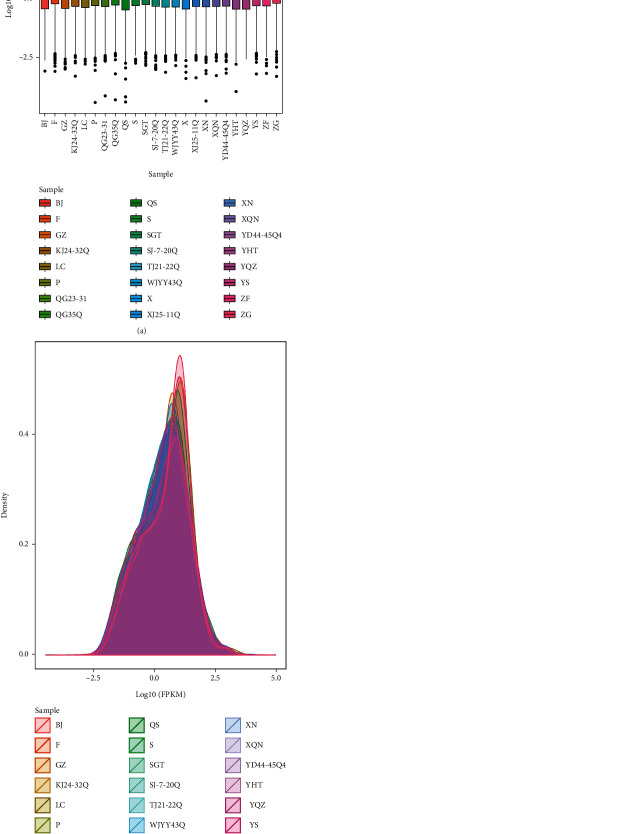
Expression levels for each sample are shown in box chart (a) and mRNAs FPKM density distribution in each sample (b).

Using RNA-seq, a sum of 26363 mRNAs transcripts were detected, including 5574 novel mRNAs transcripts. The expression of transcripts was presented by FPKM value. The discrete angle of expression levels for each sample is shown in the box chart of Figure 2(a) and the mRNAs FPKM density distribution in all samples is shown in Figure 2(b).

#### 3.4.2. Correlation Assessment of Biological Replicates

**Figure 3:**
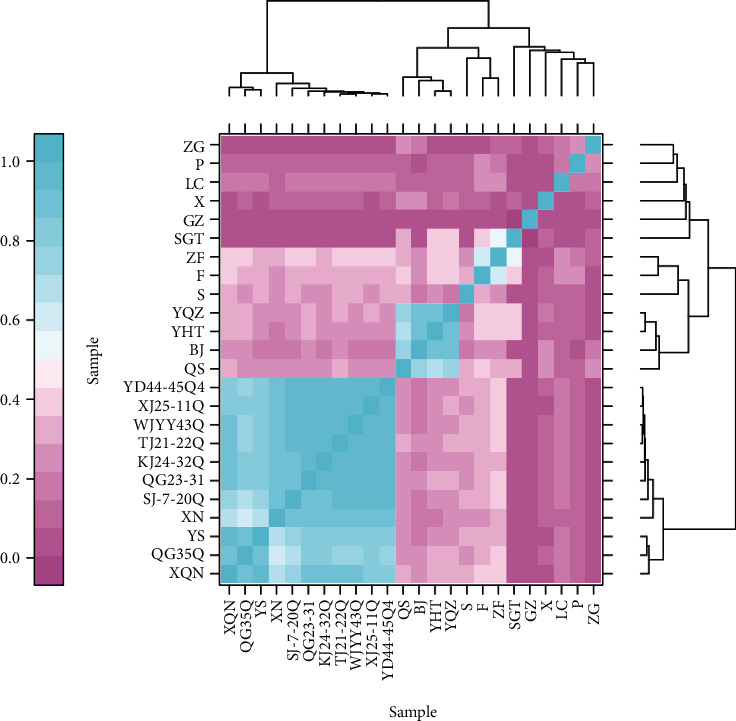
Heatmap for the correlation coefficients of samples.

For transcriptomic data of biological samples, the correlation assessment is important which could provide reliable differentially expressed genes. To evaluate the index of correlation among all the samples, we used Pearson's correlation coefficient R for multiple biological samples prepared under the same conditions. The two samples are more related to each other with *R*^2^ value close to 1 (Figure 3). So, we developed a relationship cluster diagram that reflected the relationship of the samples instinctively (Figure 3). The transcriptomic data reflected a consistent clustering effect where the samples XQN, QG35Q, YS, XN, SJ-7-20Q, QG23-31, KJ24-32Q, TJ21-22Q, WJYY43Q, XJ25-11Q, and YD44-45Q were found close to 1 and highly correlated to each other ensuring the reliability of the analysis (Figure 3).

#### 3.4.3. Identification and Statistics of Differentially Expressed Genes

**Figure 4:**
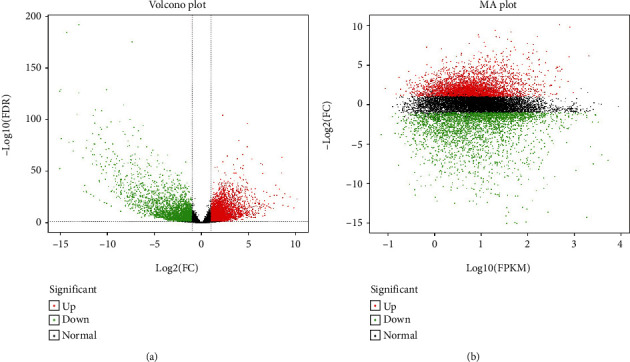
(a) Volcano plot presentation of DEGs (b) MA plot of DEGs. [Red, green, and black colors indicating the upregulated, downregulated, and normal genes, respectively].

We used False Discovery Rate (FDR) < 0.05 and Fold Change (FC) ≥ 2 value as the screening criteria to identify DEGs. The FC values specified the proportion of gene expression in two groups (brain vs. non-brain tissues). The differentially expressed genes analysis was based on independent statistical hypothesis testing, follow-on some false positives. Thus, we employed the Benjamini-Hochberg technique to correct the *P* value and made FDR a screening criterion. A total of 7,194 differentially expressed genes (DEGs) were identified, among which 3,999 were upregulated while 3,195 downregulated. The Volcano and MA plot was used for the presentation of gene expression level differences and the statistical significance in two groups (Figures 4(a) and 4(b)).

#### 3.4.4. Clustering Analysis of DEGs

**Figure 5:**
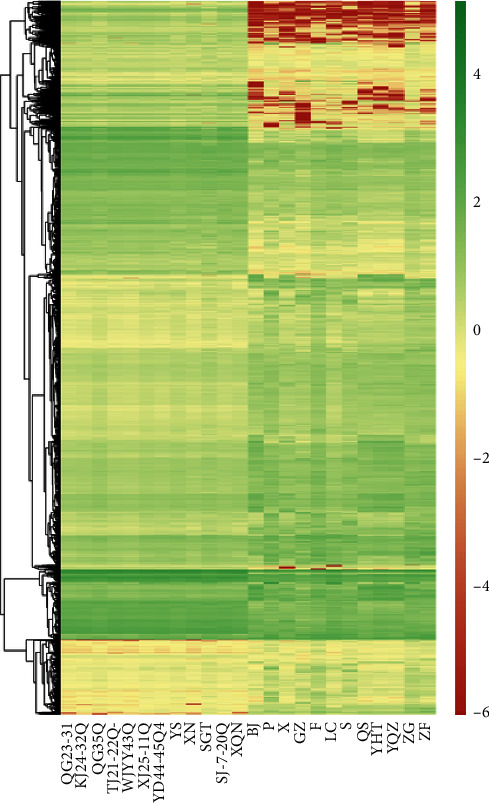
Cluster analysis of DEGs.

For hierarchical clustering analysis, the genes with differential expression were filtered and the genes with similar or same expression patterns were clustered together. The results of clustering analysis for DEGs in all the samples of both groups are shown in Figure 5.

#### 3.4.5. Functional Annotation and Enrichment Analysis of DEGs

A total of 7,121 DEGs (brain vs. non-brain) were annotated in different functional annotation databases including GO, COG, KOG, KEGG, Pfam, Swiss-Prot, eggnog, and NR with DEGs numbers 6267, 2152, 4781, 4744, 6431, 5493, 6929, and 7091, respectively.

#### 3.4.6. Gene Ontology Classification of DEGs

**Figure 6:**
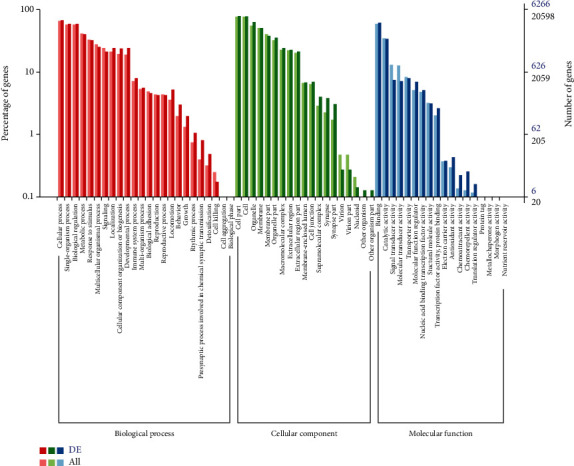
GO classification results of DEGs.

For DEGs, the GO database was used to determine their role in biological processes, cellular components, and their molecular functions (Figure 6). The cellular component related DEGs were mainly involved in the extracellular region, membrane enclosed lumen, cell junction, synapses, supramolecular complex, virion part, nucleoid, and macromolecular complexes (Figure 6). Further, the DEGs involved in the biological process were associated with gene regulation, metabolism, development, immune system, behavior, growth, locomotion, apoptosis, rhythmic process, detoxification, reproduction, response to stimulus, signal transduction processes, and cellular response to abiotic stresses (Figure 6). The molecular functions related to DEGs included metabolic, signal transduction, transportation of molecules, antioxidant activity, transcription binding factors, protein tag, morphogen activity, electron transporter, and structural and binding activity (Figure 6).

#### 3.4.7. Cluster of Orthologous Groups Analysis of DEGs

**Figure 7:**
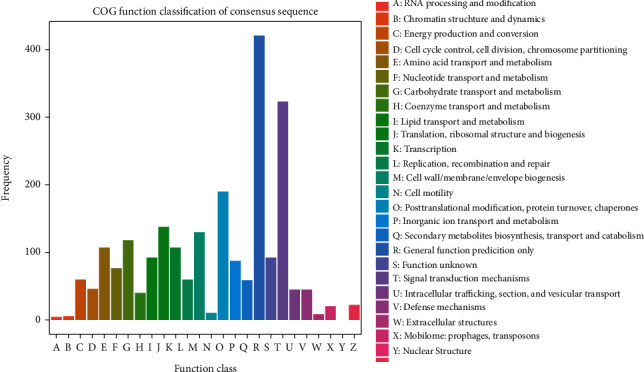
The COG annotation of DEGs.

The COG database was also used for the annotation of DEGs (Figure 7). The products of DEGs were involved in general gene function, signal transduction mechanisms, posttranslational modification, protein turnover, chaperon activity, cell motility, metabolism, transportation, cellular and nuclear structural maintenance, transportation, defense mechanisms, etc. (Figure 7).

#### 3.4.8. KEGG Annotation and Pathway Enrichment Analysis of DEGs

**Figure 8:**
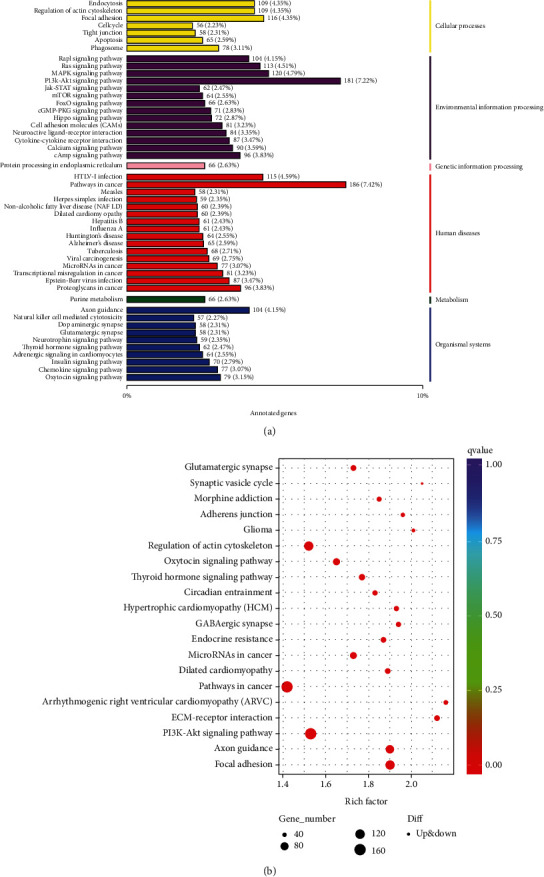
(a) KEGG annotation of DEGs. (b) The top 20 KEGG pathway enrichment bubble diagram of DEGs.

The KEGG database was used to annotate the DEGs and explore their association with different pathways. All the DEGs were classified according to their involvement in different functional pathways. About 591 DEGs were identified to be associated with pathways of cellular processes including endocytosis, regulation of actin cytoskeleton, cell cycle, apoptosis, phagosome, and tight junction, while 1291 DEGs were linked with different pathways of environmental information processing including various signaling pathways and molecular interactions (Figure 8(a)). Furthermore, for metabolism and genetic information processing related pathways, only 66 DEGs for each functional group were identified (Figure 8(a)). Moreover, the top 20 KEGG pathways with minimum *Q* values, which were analyzed by enrichment analysis for DEGs, are presented in Figure 8(b).

## 4. Discussion

The availability of massive DNA, RNA, and proteomic sequencing technologies has revolutionized the biological approaches which ultimately provides huge sequenced data output. For species with significant economic worth and poor genomic data resources like buffalo, it is imperative to develop improved and annotated sequenced genomic or transcriptome data, which would be helpful for understanding the genetic control of economic traits, genomic diversity, and evolutionary dynamics of available buffalo genetics resources. Transcriptomic sequencing is a cost-effective and powerful tool for producing good quality transcriptome data that might be used to explicate molecular markers and categorizing the novel genes in non-model and model organisms [45–48].

The advancement in this reverence requires both the data accuracy and reliability to decrease the error rate making the data more efficient [49]. Thus, using Phred quality scores *Q* with base calling error probabilities *P* is a crucial step to access the data quality [49]. A recent study by Singh et al. [50] isolated the RNA transcripts from buffalo liver tissues with an excellent quality of NGS data having score of FastQC quality up to 30. Further, they reported 54 million reads having an alignment percentage of > 84% with reference genome [50].

The 178.57 Gb clean transcriptomic data of our study with a minimum *Q* score of ≥ Q30 revealed a 1/1000 probability of incorrect base calling and the clean reads with GC contents were 28337044 and 52.77%, respectively. Further, the clean data reads without paired ends were mapped to the reference genome with an alignment percentage of 95.36%. Earlier studies have reported that the genome wide percentage of the GC-contents in river buffalo was 42.20% and in other animals, it was 41.80%–42.30% [51–54], while our study presented a higher ratio of 52.77% GC contents. Our study is also in line with a previous study conducted on swamp buffalo having GC contents of 49.92% [55]. Moreover, a recent study on the whole genome sequence of buffalo figured out the 12.5% structural differences and 9170 structural differences were likely because of the assembly errors [56].

In comparison to the reference genome, the predicted percentage of the exonic region was 88.49% and the SNPs ratio was 456941 with a higher proportion of transition as compared to the transversion with heterozygosity of 25.04%. Mostly, the detected SNPs and InDels were present in the intronic regions. This indicated the high quality transcriptome data produced from the swamp buffalo and these tissue specific unique transcripts could be utilized for designing further experiments related to transgenesis, gene cloning, and molecular genetics of the swamp buffalo [57, 58].

The alternative splicing event could produce different transcripts encoded by a single gene and can translate into protein, which varies in their sequence and function. It is an important mechanism involved in tissue specific gene expression regulation and can enhance protein diversity [59]. In this study, a total of 12 alternative splicing events were identified of which TSS, TTS, AE, and SKIP were the most abundant mapped alternative splicing events where the first alternative 5′ exon splicing and alternative 3′ last exon the last exon splicing were highly screened in all tissue samples.

We identified a total of 26363 mRNAs transcripts including 5574 novel mRNAs transcripts. Our study presented 34.57% novel genes whose function has not been yet identified after blasting all these novel genes in different databases including GO, KEGG, KOG, Pfam, Swiss-Prot, egg NOG, NR, and COG for annotated information, while the previous study indicated only 27.53% novel gene in swamp buffalo with unknown function [55]. For any transcriptomic analysis, the identification of DEGs is very critical [60]. Our study found variations in the expression of genes such as about 55.58% (3,999) genes were upregulated and 44.41% (3,195) were downregulated genes. In the NR database, most of the genes were annotated to *Bubalus bubalis*, *Bos taurus,* and *Bos mutus*, perhaps due to the swamp buffalo is evolutionary more close to *Bos taurus* and *Bos mutus* than other available genetic resources [61, 62].

The GO and COG predicted molecular functioning of DEGs as metabolic, signal transduction, electron transporter, and structural and binding activity. Moreover, among the top 20 pathways, cancer pathway, PI3k-Akt signaling, axon guidance, focal adhesion, and regulation of actin cytoskeleton were abundant while the other related pathways were involved in thyroid and oxytocin hormone signaling, synaptic vesicle cycle, adhere junction, circadian entertainment, etc. These findings are in agreement with the previous study of Deng et al. [55].

## 5. Conclusion

The current study is one of the most comprehensive studies conducted on the swamp buffalo using 24 tissue samples, which were grouped into two main categories (brain vs. non-brain). We obtained 178.57 Gb clean transcriptome data with *Q*score ≥ *Q*30 and the clean reads with GC contents were 28337044 and 52.77%, respectively. The alignment percentage of clean data reads to the reference genome was 95.36% with 88.49% exon region coverage, and the SNPs ratio was 456941 with higher transition SNPs proportion with 25.04% heterozygosity. We incur 26363 mRNAs transcripts including 5574 (34.57%) novel genes of which 55.58% (3,999) were upregulated and 44.41% (3,195) downregulated. The DEGs were mainly involved in the metabolism, signal transduction, electron transporter, structural and binding activities, among the top hit pathways, cancer pathway, PI3k-Akt signaling, axon guidance, focal adhesion, and regulation of actin cytoskeleton were abundant.

## Figures and Tables

**Table 1:** Details of tissues of swamp buffalo used for sample collection.

Sr. no.	Sample tissue	Abbreviation
1	Dorsal muscles	BJ
2	Lung	F
3	Liver	GZ
4	Pain sense(24 + 32 area)	KJ24-32Q
5	Oarium (ovarium or ovary)	LC
6	Spleen	P
7	Emotional area 23 + 31	QG23-31
8	Emotional area 35	QG35Q
9	Anterior tongue muscle	QS
10	Kidney	S
11	Conarium	SGT
12	Visual sense (7-20 area)	SJ-7-20Q
13	Sense of hearing (21-22 area)	TJ21-22Q
14	Taste, language 43 area	WJYY43Q
15	Heart	X
16	Sense of smell (25 + 11 area)	XJ25-11Q
17	Opisthencephalon	XN
18	Hypothalamus	XQN
19	Sport (44-45 area, 4 + 6 area)	YD44-45Q
20	Right hind leg muscle	YHT
21	Right fore-muscle	YQZ
22	Bulbus rhachidicus	YS
23	Fattiness	ZF
24	Uterus	ZG

**Table 2:** The summary of novel gene number annotated in different databases.

Annotated databases	Novel gene number
GO	2,629
KEGG	1,713
KOG	541
Pfam	719
Swiss-Prot	753
Eggnog	2,218
NR	3,619
COG	82
All	3,647

## Data Availability

The transcriptome sequences data of the swamp buffalo 24 tissues (12 brain and 12 non-brain tissues) were deposited to the NCBI-GenBank BioProject under the accession number PRJNA760646.

## Supplementary Materials

Supplementary Materials Figure S1. The data quality of the samples with ATCG content distribution and base error rates. Figure S2. Position of mapped reads on the reference genome and coverage depth distribution. Figure S3. (a) The depth distributions of mapped reads on transcripts (b) the RNA-seq data saturation simulation. Figure S4. The types of SNP mutations in sequenced data (transition and transversion). Figure S5. The genes SNP density distribution. Figure S6. Statistics of alternative splicing events. Table S1. The statistics of the sequenced data. Table S2. The sequenced data mapping statistics. Table S3. Percentage of exonic, intergenic, and intronic regions of mapped reads on the reference genome. Table S4. SNP statistics detected in all samples.
