# Supplementary material for: De Novo Transcriptome Dataset Generation of the Swamp Buffalo Brain and Non-Brain Tissues
Source: Biomed Res Int. 2022 Oct 12;2022:4472940. doi: 10.1155/2022/4472940 (PMC9668446; doi:10.1155/2022/4472940)
Supplement: Supplementary Materials — Figure S1. The data quality of the samples with ATCG content distribution and base error rates. Figure S2. Position of mapped reads on the reference genome and coverage depth distribution. Figure S3. (a) The depth distributions of mapped reads on transcripts (b) the RNA-seq data saturation simulation. Figure S4. The types of SNP mutations in sequenced data (transition and transversion). Figure S5. The genes SNP density distribution. Figure S6. Statistics of alternative splicing events. Table S1. The statistics of the sequenced data. Table S2. The sequenced data mapping statistics. Table S3. Percentage of exonic, intergenic, and intronic regions of mapped reads on the reference genome. Table S4. SNP statistics detected in all samples. [file 4472940.f1.pdf]

__Supplementary material__

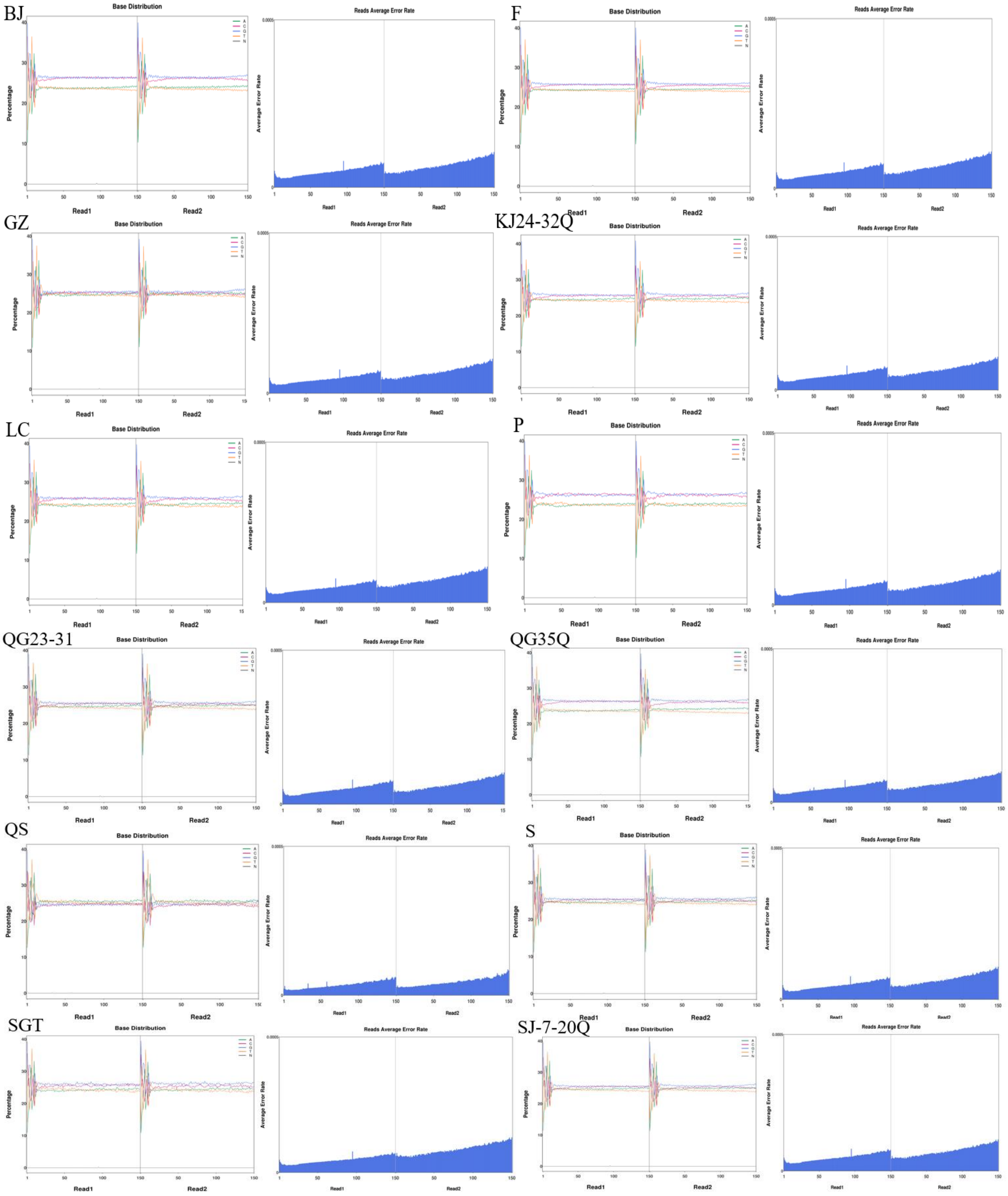

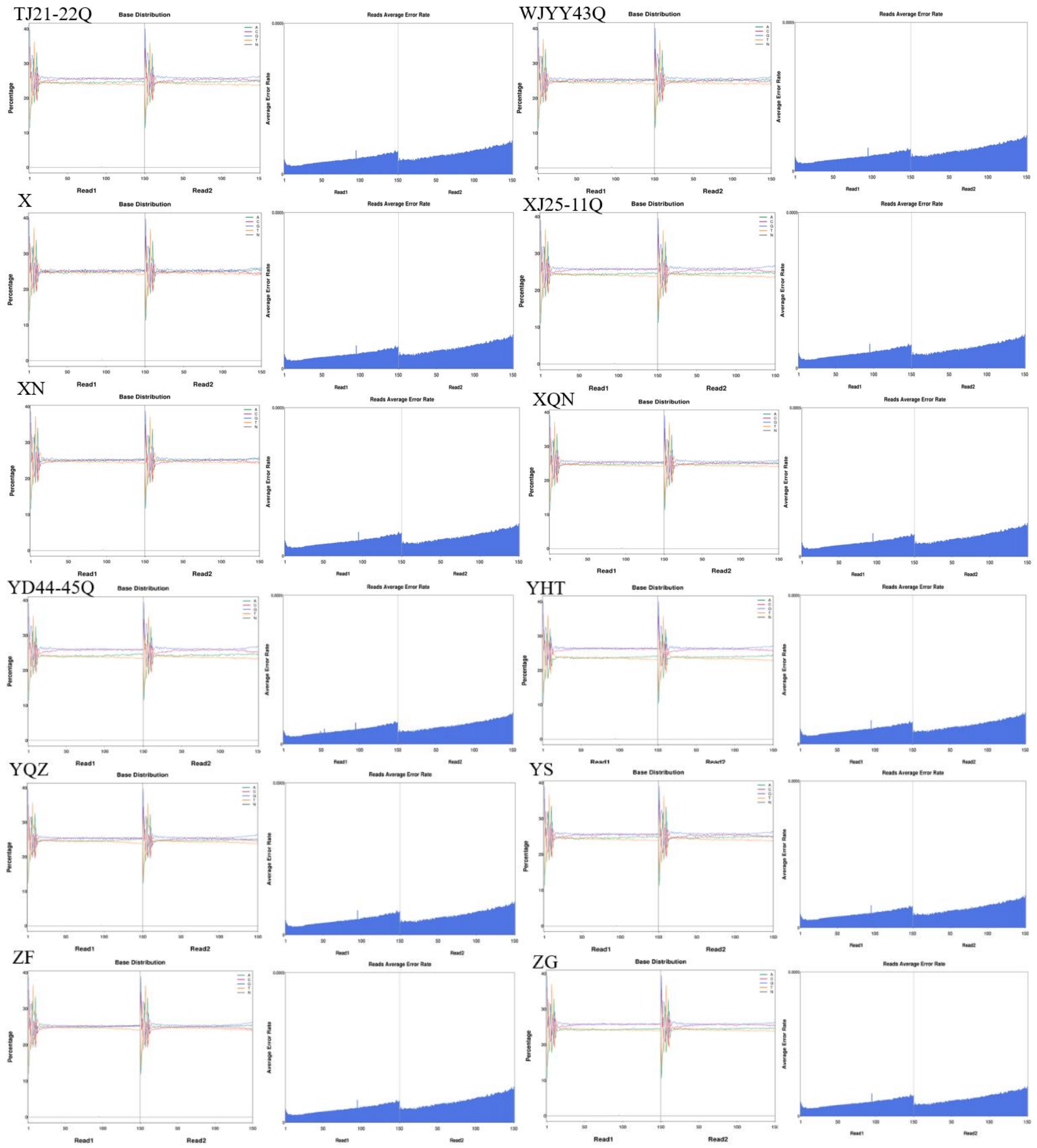

Figure S1. The data quality of the samples with ATCG content distribution and base error rates.

[Note: abscissa is the base position of a read, the ordinate is the proportion of base and average error rate for the single base].

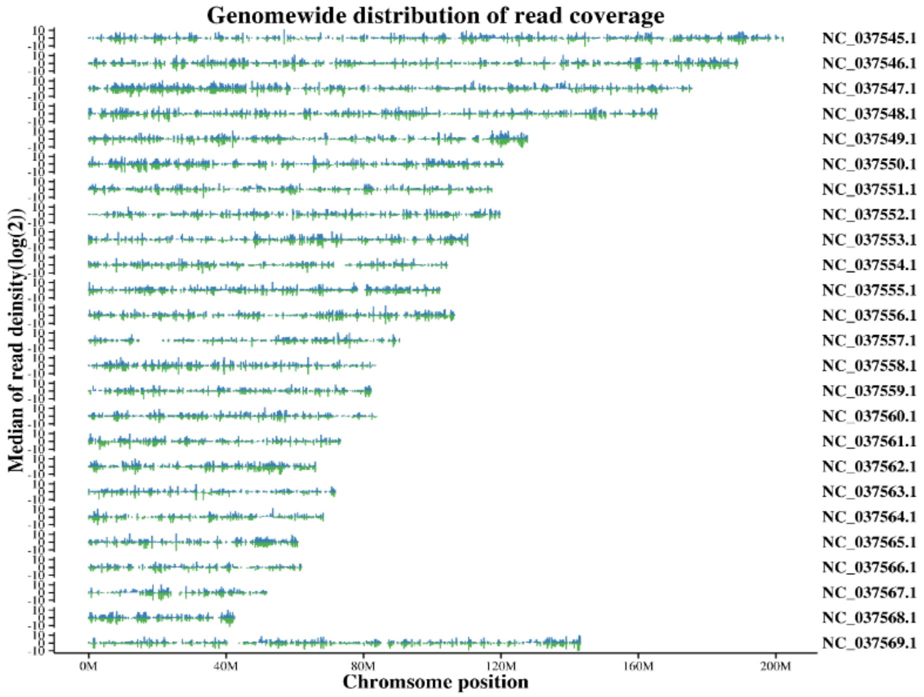

Figure S2. Position of mapped reads on the reference genome and coverage depth distribution.

(A)

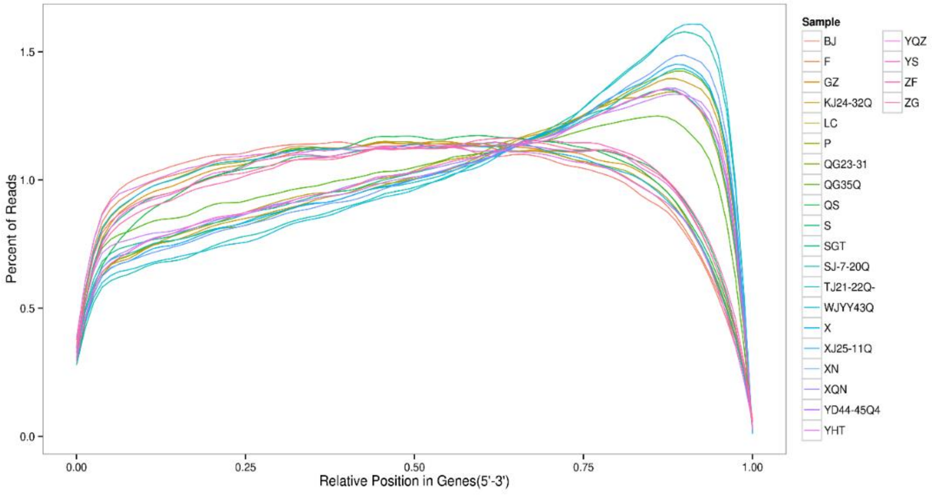

(B)

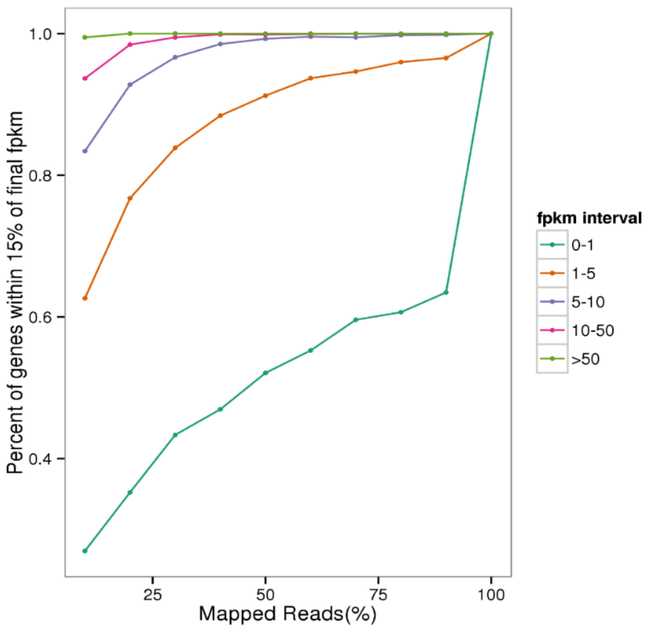

Figure S3. (A) The depth distributions of mapped reads on transcripts **(B)** the RNA-seq data saturation simulation.

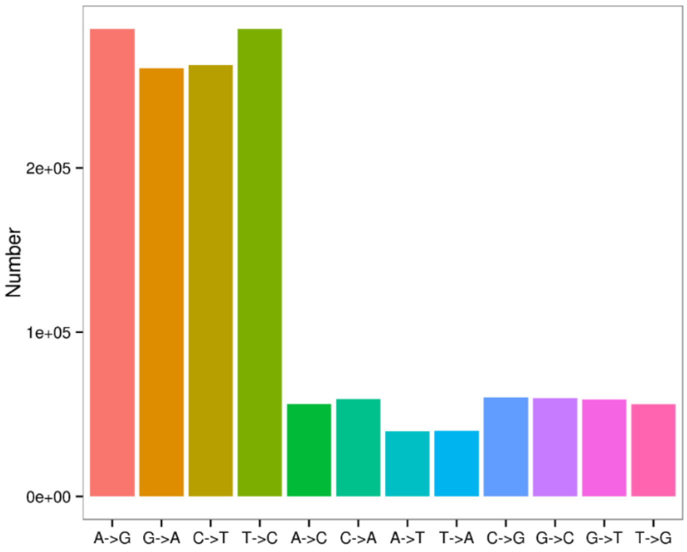

Figure S4. The types of SNP mutations in sequenced data (transition and transversion).

[Note: The horizontal axis represents the SNP mutation types, the vertical axis represents the number of the corresponding SNPs].

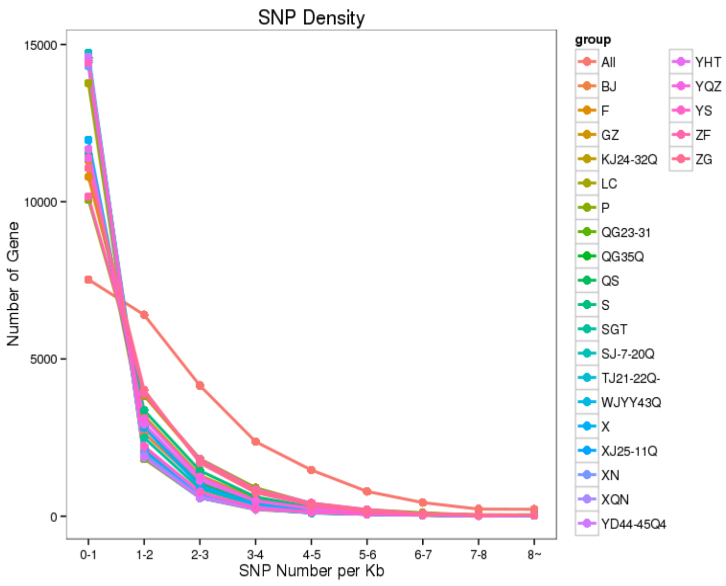

Figure S5. The genes SNP density distribution.

[Note: The abscissa represents the average number of SNPs distribution per 1000bp gene sequence, the ordinate represents the number of genes.]

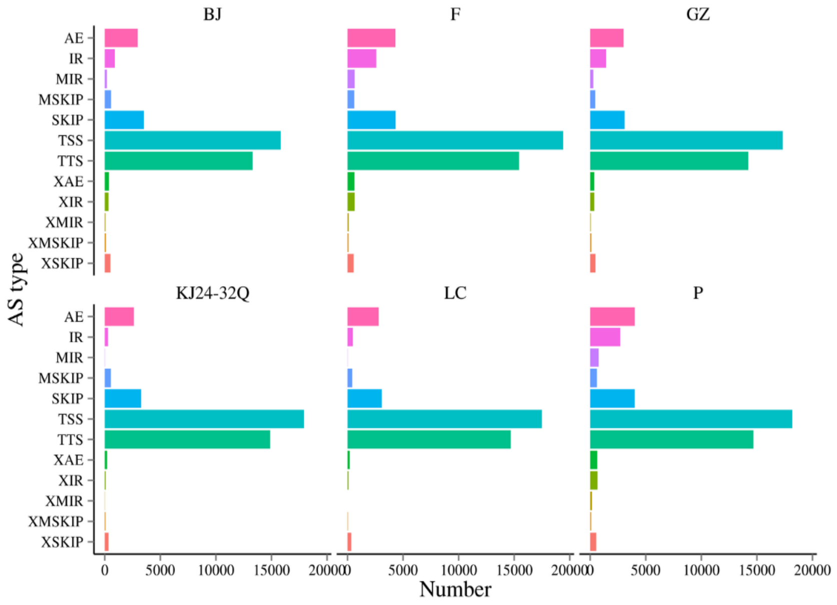

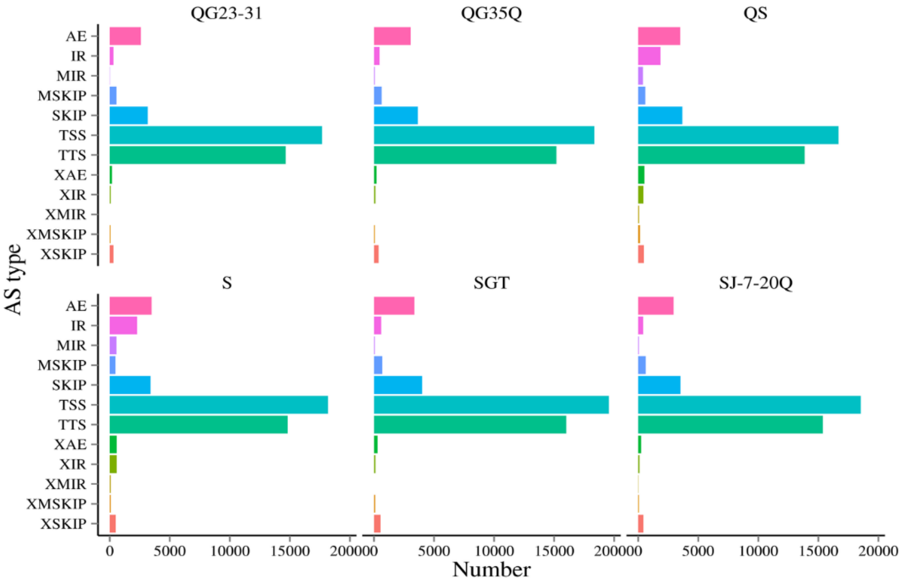
QG23-31
QG35Q
QS
S
SGT
SJ-7-20Q
AE
IR
MIR
MSKIP
SKIP
TSS
TTS
XAE
XIR
XMIR
XMSKIP
XSKIP
AS type
0
5000
10000
15000
20000
Number

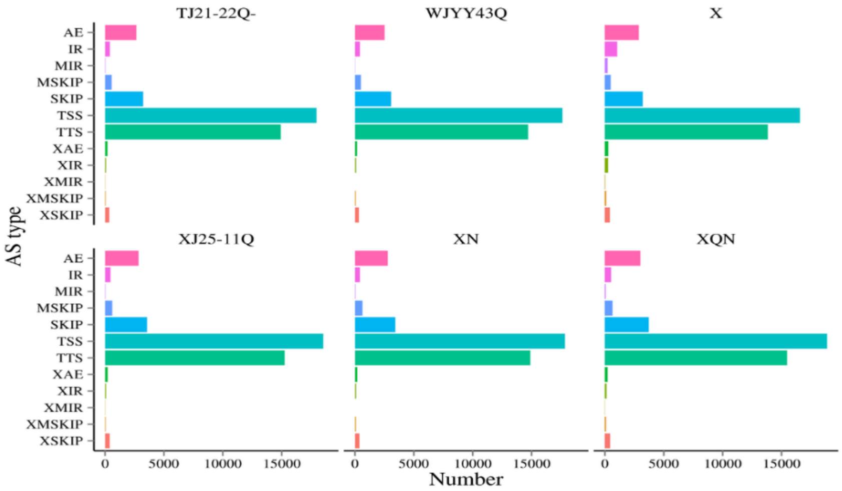
TJ21-22Q-
WJYY43Q
X
XJ25-11Q
XN
XQN
AE
IR
MIR
MSKIP
SKIP
TSS
TTS
XAE
XIR
XMIR
XMSKIP
XSKIP
AS type
0
5000
10000
15000
Number

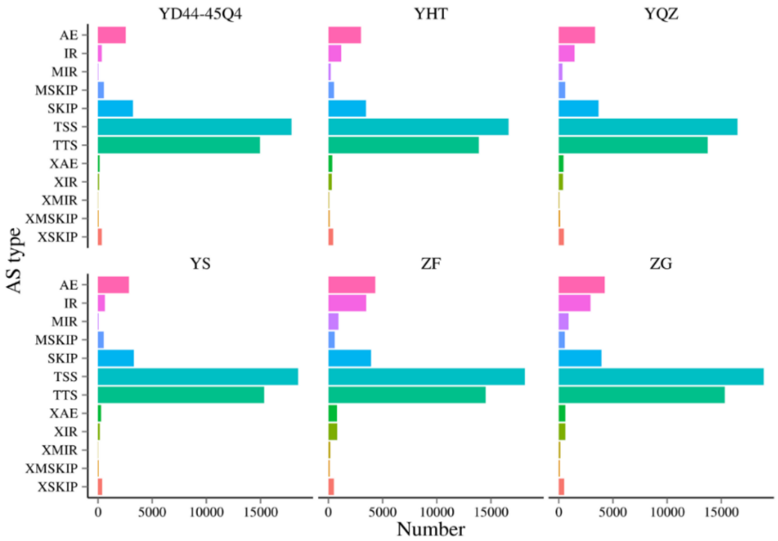

Figure S6. Statistics of alternative splicing events.

[Note: The horizontal axis represents a number to one of the alternative transcripts, the vertical axis represents types of alternative splicing events.1-TSS: Alternative 5' first exon (transcription start site) the first exon splicing; 2-TTS: Alternative 3' last exon (transcription terminal site) the last exon splicing; 3-SKIP: Skipped exon (SKIP_ON, SKIP_OFF pair) single exon skipping; 4-XSKIP: Approximate SKIP (XSKIP_ON, XSKIP_OFF pair) single exon skipping (fuzzy boundary); 5-MSKIP: Multi-exon SKIP (MSKIP_ON, MSKIP_OFF pair) multi-exon skipping; 6-XMSKIP: Approximate MSKIP (XMSKIP_ON, XMSKIP_OFF pair) multi-exon skipping (fuzzy boundary); 7-IR: Intron retention (IR_ON, IR_OFF pair) single intron retention; 8-XIR: Approximate IR (XIR_ON, XIR_OFF pair) single intron retention (fuzzy boundary); 9-MIR: Multi-IR (MIR_ON, MIR_OFF pair) multi-intron retention; 10-XMIR: Approximate MIR (XMIR_ON, XMIR_OFF pair) multi-intron retention (fuzzy boundary); 11-AE: Alternative exon ends (5', 3', or both); 12-XAE: Approximate AE variable 5' or 3' end (fuzzy boundary)].

Table S1. The statistics of the sequenced data.

**Samples**	**Clean reads**	**Clean bases**	**GC Count**	**%≥Q30**
BJ	21,868,067	6,560,420,100	52.67%	92.42%
F	25,386,599	7,615,979,700	51.46%	92.29%
GZ	26,166,123	7,849,836,900	50.63%	92.34%
KJ24-32Q	23,320,858	6,996,257,400	51.44%	92.17%
LC	27,749,317	8,324,795,100	51.79%	92.07%
P	26,472,161	7,941,648,300	52.63%	92.52%
QG23-31	23,323,713	6,997,113,900	51.05%	92.33%
QG35Q	21,971,799	6,591,539,700	52.68%	92.62%
QS	28,337,044	8,501,113,200	49.47%	94.61%
S	22,618,701	6,785,610,300	50.87%	92.29%
SGT	27,371,168	8,211,350,400	51.66%	91.27%
SJ-7-20Q	28,331,213	8,499,363,900	51.07%	91.84%
TJ21-22Q-	24,040,796	7,212,238,800	51.37%	91.98%
WJYY43Q	24,677,989	7,403,396,700	50.76%	91.77%
X	25,449,910	7,634,973,000	50.46%	92.25%
XJ25-11Q	24,869,758	7,460,927,400	51.57%	92.19%
XN	26,251,060	7,875,318,000	50.39%	92.06%
XQN	24,571,518	7,371,455,400	50.82%	92.11%
YD44-45Q4	22,511,689	6,753,506,700	52.05%	92.59%
YHT	23,406,717	7,022,015,100	52.77%	92.28%
YQZ	25,582,045	7,674,613,500	50.92%	92.20%
YS	23,182,550	6,954,765,000	51.21%	92.26%
ZF	25,817,126	7,745,137,800	50.48%	92.10%
ZG	21,970,637	6,591,191,100	51.56%	92.46%

[Note: Samples: sample name; Clean reads: Paired-end numbers of Clean Data; Clean bases: total base number of Clean Data; GC content: GC content percentage of Clean Data, namely the percentage of Clean Database G and C; ≥Q30%: the base which quality value is greater than or equal to 30 percentage of Clean Data].

Table S2. The sequenced data mapping statistics.

Sample	Total Reads	Mapped Reads	Unique Mapped Reads	Multiple Map Reads	Reads Map to (+)	Reads Map to (-)
BJ	43,736,134	41,462,810 (94.80%)	40,299,471 (92.14%)	1,163,339 (2.66%)	20,637,894 (47.19%)	20,661,919 (47.24%)
F	50,773,198	47,925,692 (94.39%)	46,594,374 (91.77%)	1,331,318 (2.62%)	23,842,833 (46.96%)	23,889,168 (47.05%)
GZ	52,332,246	49,731,001 (95.03%)	47,888,782 (91.51%)	1,842,219 (3.52%)	24,736,589 (47.27%)	24,804,154 (47.40%)
KJ24-32Q	46,641,716	44,355,163 (95.10%)	43,052,932 (92.31%)	1,302,231 (2.79%)	21,990,611 (47.15%)	22,066,742 (47.31%)
LC	55,498,634	52,015,315 (93.72%)	49,334,684 (88.89%)	2,680,631 (4.83%)	25,859,450 (46.59%)	25,785,017 (46.46%)
P	52,944,322	47,443,076 (89.61%)	44,938,700 (84.88%)	2,504,376 (4.73%)	23,671,040 (44.71%)	23,184,563 (43.79%)
QG23-31	46,647,426	44,247,268 (94.85%)	43,033,828 (92.25%)	1,213,440 (2.60%)	21,923,341 (47.00%)	21,997,123 (47.16%)
QG35Q	43,943,598	41,748,439 (95.00%)	40,461,427 (92.08%)	1,287,012 (2.93%)	20,689,864 (47.08%)	20,773,280 (47.27%)
QS	56,674,088	50,738,692 (89.53%)	49,403,052 (87.17%)	1,335,640 (2.36%)	25,265,088 (44.58%)	25,208,609 (44.48%)
S	45,237,402	42,974,783 (95.00%)	41,801,772 (92.41%)	1,173,011 (2.59%)	21,382,931 (47.27%)	21,422,436 (47.36%)
SGT	54,742,336	51,760,825 (94.55%)	49,866,550 (91.09%)	1,894,275 (3.46%)	25,642,929 (46.84%)	25,735,273 (47.01%)
SJ-7-20Q	56,662,426	53,600,479 (94.60%)	52,158,660 (92.05%)	1,441,819 (2.54%)	26,593,449 (46.93%)	26,655,872 (47.04%)
TJ21-22Q-	48,081,592	45,672,174 (94.99%)	44,346,935 (92.23%)	1,325,239 (2.76%)	22,636,452 (47.08%)	22,708,367 (47.23%)
WJYY43Q	49,355,978	46,817,969 (94.86%)	45,645,423 (92.48%)	1,172,546 (2.38%)	23,275,082 (47.16%)	23,287,090 (47.18%)
X	50,899,820	48,428,087 (95.14%)	47,343,162 (93.01%)	1,084,925 (2.13%)	24,176,677 (47.50%)	24,162,981 (47.47%)
XJ25-11Q	49,739,516	46,820,454 (94.13%)	45,374,560 (91.22%)	1,445,894 (2.91%)	23,212,367 (46.67%)	23,274,660 (46.79%)
XN	52,502,120	49,968,072 (95.17%)	48,583,120 (92.54%)	1,384,952 (2.64%)	24,817,682 (47.27%)	24,847,822 (47.33%)
XQN	49,143,036	46,862,566 (95.36%)	45,559,665 (92.71%)	1,302,901 (2.65%)	23,238,763 (47.29%)	23,307,461 (47.43%)
YD44-45Q4	45,023,378	42,562,217 (94.53%)	41,196,162 (91.50%)	1,366,055 (3.03%)	21,090,218 (46.84%)	21,156,215 (46.99%)

续上表

YHT	46,813,434	44,161,365 (94.33%)	42,794,150 (91.41%)	1,367,215 (2.92%)	21,972,723 (46.94%)	21,998,425 (46.99%)
YQZ	51,164,090	47,976,420 (93.77%)	46,582,386 (91.05%)	1,394,034 (2.72%)	23,837,781 (46.59%)	23,882,301 (46.68%)
YS	46,365,100	43,731,874 (94.32%)	42,463,219 (91.58%)	1,268,655 (2.74%)	21,711,132 (46.83%)	21,764,078 (46.94%)
ZF	51,634,252	48,206,664 (93.36%)	46,979,834 (90.99%)	1,226,830 (2.38%)	23,894,475 (46.28%)	23,999,694 (46.48%)
ZG	43,941,274	41,592,901 (94.66%)	40,530,999 (92.24%)	1,061,902 (2.42%)	20,616,304 (46.92%)	20,673,396 (47.05%)

[Note: Samples: sample name; Total Reads: Reads number of Clean Data, not paired-end reads; Mapped Reads: Reads Number Mapped to the reference genome and the percentage in Clean Reads; Unique Mapped Reads: Reads Number Mapped uniquely mapped to the reference genome and the percentage in Clean Reads; Multiple Mapped Reads: Reads number multiply mapped to reference genome and the percentage in Clean Reads; Reads Map to (+): Reads number mapped to the sense chain and the percentage in Clean Reads. Reads Map to (-): Reads number mapped to the antisense chain and the percentage in Clean Reads].

Table S3. Percentage of exonic, intergenic, and intronic regions of mapped reads on the reference genome.

**Sample**	**Exon**	**Intergenic**	**Intron**
BJ	83.47%	6.43%	10.10%
F	78.75%	6.86%	14.57%
GZ	82.38%	6.04%	11.58%
KJ24-32Q	87.63%	7.26%	5.11%
LC	88.49%	6.46%	5.05%
P	76.56%	7.32%	16.12%
QG23-31	87.39%	7.38%	5.23%
QG35Q	87.08%	7.36%	5.56%
QS	80.10%	7.72%	12.18%
S	78.45%	7.36%	14.19%
SGT	85.92%	7.86%	6.22%
SJ-7-20Q	87.20%	7.45%	5.35%

TJ21-22Q-	87.32%	7.18%	5.50%
WJYY43Q	87.24%	7.49%	5.27%
X	82.70%	7.06%	10.24%
XJ25-11Q	86.90%	7.46%	5.64%
XN	87.29%	7.33%	5.38%
XQN	86.40%	7.66%	5.94%
YD44-45Q4	87.97%	6.79%	5.24%
YHT	83.68%	6.40%	9.92%
YQZ	81.62%	6.87%	11.51%
YS	86.48%	7.23%	6.29%
ZF	75.81%	7.45%	16.74%
ZG	77.09%	7.89%	15.02%

Table S4. SNP statistics detected in all samples.

Sample	SNP Number	Genic SNP	Intergenic SNP	Transition	Transversion	Heterozygosity
BJ	305,396	264,414	40,982	72.57%	27.43%	20.62%
F	494,560	433,053	61,507	72.18%	27.82%	21.65%
GZ	360,607	321,024	39,583	72.55%	27.45%	21.19%
KJ24-32Q	189,058	155,893	33,165	73.01%	26.99%	25.04%
LC	193,900	164,494	29,406	73.08%	26.92%	24.78%
P	495,289	440,056	55,233	72.12%	27.88%	21.50%
QG23-31	189,382	154,890	34,492	72.79%	27.21%	24.71%
QG35Q	209,565	174,205	35,360	73.22%	26.78%	23.95%
QS	339,545	295,807	43,738	72.11%	27.89%	20.31%
S	413,458	362,909	50,549	72.42%	27.58%	20.63%
SGT	258,726	209,218	49,508	72.48%	27.52%	23.95%
SJ-7-20Q	215,004	174,830	40,174	72.83%	27.17%	24.59%
TJ21-22Q-	191,806	158,908	32,898	72.92%	27.08%	24.44%
WJYY43Q	188,314	154,813	33,501	73.03%	26.97%	24.57%

X	304,751	266,582	38,169	72.54%	27.46%	21.09%
XJ25-11Q	213,327	176,274	37,053	72.98%	27.02%	24.22%
XN	205,645	166,926	38,719	72.75%	27.25%	24.66%
XQN	226,332	186,483	39,849	72.78%	27.22%	23.85%
YD44-45Q4	183,666	153,151	30,515	73.03%	26.97%	24.67%
YHT	316,166	276,746	39,420	72.70%	27.30%	20.82%
YQZ	360,544	315,887	44,657	72.28%	27.72%	20.71%
YS	223,159	187,640	35,519	72.86%	27.14%	23.50%
ZF	525,235	456,941	68,294	71.94%	28.06%	21.44%
ZG	479,652	418,661	60,991	72.17%	27.83%	21.69%

[Note: Samples: sample name; SNP Number: total numbers of SNPs; Genic SNP: total numbers of SNPs in the genic region; Intergenic SNP: total numbers of SNPs between genes; Transition: the percentage that the transition-type SNP accounts for all SNP loci's; Transversion: the percentage that the transversion-type SNP loci account for all SNP sites; Heterozygosity: the percentage that the heterozygous SNPs account for all SNPs].
